# Molecular characteristics and pathogenic mechanisms of KPC-3 producing hypervirulent carbapenem-resistant *Klebsiella pneumoniae* (ST23-K1)

**DOI:** 10.3389/fcimb.2024.1407219

**Published:** 2024-08-15

**Authors:** Yanye Tu, Hui Gao, Rongqing Zhao, Jiliang Yan, Xingbing Wu

**Affiliations:** Department of Clinical Laboratory, The Affiliated Li Huili Hospital, Ningbo University, Ningbo, China

**Keywords:** hypervirulent carbapenem-resistant *Klebsiella pneumoniae*, pLVPK-like virulence plasmid, bla KPC-3 resistance plasmid, resistance genes, whole-genome sequencing

## Abstract

**Objective:**

This study aimed to comprehensively investigate hypervirulent carbapenem-resistant *Klebsiella pneumoniae* (CR-hvKP) in the Ningbo region. Importantly, we sought to elucidate its molecular characteristics and pathogenic mechanisms. This information will provide evidence-based insights for preventing and controlling nosocomial infections and facilitate improved clinical diagnosis and treatment in this region.

**Methods:**

96 carbapenem-resistant *Klebsiella pneumoniae* strains were collected from the Ningbo region between January 2021 and December 2022. Whole genome sequencing and bioinformatic methods were employed to identify and characterize CR-hvKP strains at the molecular level. The minimum inhibitory concentrations (MICs) of common clinical antibiotics were determined using the VITEK-2 Compact automatic microbiological analyzer. Plasmid conjugation experiments evaluated the transferability of resistance plasmids. Finally, mouse virulence assays were conducted to explore the pathogenic mechanisms.

**Results:**

Among the 96 strains, a single CR-hvKP strain, designated CR-hvKP57, was identified, with an isolation frequency of 1.04%. Whole-genome sequencing revealed the strain to be ST23 serotype with a K1 capsule. This strain harbored three plasmids. Plasmid 1, a pLVPK-like virulence plasmid, carried multiple virulence genes, including *rmpA*, *rmpA2*, *iroB*, *iucA*, and *terB*. Plasmid 2 contained transposable element sequences such as IS15 and IS26. Plasmid 3, classified as a resistance plasmid, harbored the *bla*_KPC-3_ carbapenem resistance gene. Mouse virulence assays demonstrated a high mortality rate associated with CR-hvKP57 infection. Additionally, there was a significant increase in IL-1β, IL-6, and TNF-α levels in response to CR-hvKP57 infection, indicating varying degrees of inflammatory response. Western blot experiments further suggested that the pathogenic mechanism involves activation of the NF-κB signaling pathway.

**Conclusion:**

This study confirms the emergence of hypervirulent CR-hvKP in the Ningbo region, which likely resulted from the acquisition of a pLVPK-like virulence plasmid and a *bla*_KPC-3_ resistance plasmid by the ST23-K1 type *Klebsiella pneumoniae*. Our findings highlight the urgent need for more judicious use of antibiotics to limit the emergence of resistance. Additionally, strengthening infection prevention and control measures is crucial to minimize the spread of virulence and resistance plasmids.

## 1 Introduction

The hypervirulent CR-hvKP (hypervirulent carbapenem-resistant *Klebsiella pneumoniae*) was first identified in 1986 by the Taiwanese scholar Liu et al (Liu et al., 1986). Unlike conventional *Klebsiella pneumoniae* (cKP), hvKP exhibits stronger virulence characteristics and invasiveness, including the production of copious amounts of capsular polysaccharide to resist phagocytosis by neutrophils, leading to severe invasive infections (Chen and Chen, 2021). Common infections include liver abscesses, ocular abscesses, purulent meningitis, and osteomyelitis (Hassanin et al., 2021; Kubota et al., 2021; Liu et al., 2022a). Previous studies have suggested that hypervirulent *Klebsiella pneumoniae* with high-virulence genes tend to have low drug resistance. However, in recent years, an increasing number of studies have reported the identification of CR-hvKP. In southern India, blood samples from hospitalized patients yielded nine ST2096 CR-hvKP strains predominantly carrying *bla*_NDM-5_ and *bla*_OXA-232_ carbapenem-resistant genes (Shankar et al., 2022). Since 2016, the clonal dissemination of the hypervirulent ST147 CR-hvKP strain with hybrid resistance and virulence plasmids IncFIB/IncHI1B has been observed in 19 hospital laboratories in the UK (Turton et al., 2018, 2024). Additionally, a highly virulent clone of CR-hvKP serotype ST11-K47 was isolated from a 60-year-old female patient admitted to a tertiary hospital in Egypt (Ahmed et al., 2021). Furthermore, the ST11-K64 lineage represents the predominant CR-hvKP type in China and is widely distributed internationally (Baraniak et al., 2019; Zhou et al., 2023; Chen et al., 2023a; Wang et al., 2024). It is now understood that these strains exhibit high virulence and high drug resistance characteristics that often lead to high mortality (Wong et al., 2018; Xie et al., 2021). In this respect, five strains of CR-hvKP carrying pLVPK-like virulence plasmid were recently documented in Hangzhou, Zhejiang Province, which exhibited high virulence, drug resistance, and invasiveness (Gu et al., 2018). These types of “super strain” pose significant challenges to clinical treatment and infection prevention and control (Lee et al., 2017; Zhou et al., 2021a). Current research has revealed that the development of CR-hvKP strains involves two mechanisms. Firstly, CR-KP strains, such as ST11-K64 CRKP, can acquire virulence plasmids. For example, the pk2044-like virulence plasmid from ST23-K1 hv-KP can reportedly contribute to the emergence of ST11-K64 CR-hvKP (Wang et al., 2024). Secondly, hv-KP strains acquire carbapenem resistance plasmids from the external environment, such as *bla*_KPC-2_ becoming CR-hvKP (Zhang et al., 2024). To our knowledge, the prevalence of CR-hvKP in the Ningbo area has been largely understudied. Our research group previously identified evidence of clonal transmission within ST15 CR-KP; some strains harbored highly virulent genes, indicating a possible evolution toward CR-hvKP (Gao et al., 2024). Therefore, it is urgent to determine whether other types of hypervirulent *Klebsiella pneumoniae* exist in Ningbo and the underlying mechanism. In this study, 96 strains of carbapenem-resistant *Klebsiella pneumoniae* were screened for the presence of CR-hvKP in the Ningbo area from January 2021 to December 2022, and its molecular characteristics and pathogenic mechanism were studied, providing a theoretical basis for the prevention, minimize the concurrent transmission of virulence plasmids and resistant plasmids and control of hospital infection and clinical treatment in the local district.

## 2 Materials and methods

### 2.1 Materials

#### 2.1.1 Source and the identification of strains

Ninety-six carbapenem-resistant *Klebsiella pneumoniae* strains were collected in the Ningbo area from January 2021 to December 2022. Inclusion criteria were as follows: strains were identified as carbapenem-resistant based on the M100-S30 standard of the Clinical and Laboratory Standards Institute (CLSI) (Institute. CaLS, 2020). Duplicate strains isolated from the same patient were excluded. Bacterial identification was performed using both the VITEK 2 Gram-negative (GN) microbial identification test cards (bioMérieux, France) and the MALDI-TOF microbial mass spectrometry detection system EXS 3600 (Zhongyuan Huiji).

#### 2.1.2 Instruments and reagents

The following instruments were used in this study: VITEK 2 Compact automated microbiology system (bioMérieux, France) and EXS3600 automatic microbial mass spectrometry system (Zhongyuan Huiji). Microbroth susceptibility panels were obtained from Zhejiang Wenzhou Kante Biotechnology Co., Ltd. Quality control strains were employed: *Escherichia coli* ATCC 25922 (antibiotic susceptibility testing control) and *Klebsiella pneumoniae* ATCC 700603 (toxicity testing control), kindly provided by the Ningbo Clinical Testing Center. Hypervirulent *Klebsiella pneumoniae* NTUH-K2044 and *Escherichia coli* EC600 were procured from Hangzhou Hongsa Biotech Co., Ltd.

### 2.2 Methods

#### 2.2.1 Antimicrobial susceptibility testing

The minimum inhibitory concentrations (MICs) of commonly used antimicrobial agents against the bacteria were determined using the VITEK 2 Compact automated microbiology system. This system tested aztreonam, ceftriaxone, cefepime, ciprofloxacin, levofloxacin, imipenem, ertapenem, piperacillin/tazobactam, trimethoprim/sulfamethoxazole, nitrofurantoin, amikacin, gentamicin, and tobramycin. MICs for ceftazidime-avibactam and tigecycline were determined using the broth microdilution method. The broth microdilution method was performed as follows: Fresh colonies from an overnight culture were adjusted to a 0.5 McFarland standard turbidity. A 10 μL aliquot of the bacterial suspension was then transferred to 2 mL of Mueller-Hinton broth and mixed thoroughly. Subsequently, 100 μL of the diluted bacterial solution was transferred to the susceptibility testing plates. Plates were incubated overnight at 37°C. MICs were determined based on the absence or presence of bacterial growth in the wells. Tigecycline results were interpreted according to the U.S. Food and Drug Administration Testing standards (Testing TECoAS, 2022), while MICs for all other antimicrobials were interpreted according to the CLSI 2020 guidelines (Institute. CaLS, 2020).

#### 2.2.2 Plasmid conjugation experiment

Rifampicin-resistant *Escherichia coli* strain EC600 was used as the recipient strain for conjugation experiments. Both donor and recipient strains were cultured on Columbia agar plates. Cultures were then adjusted to a 0.5 McFarland standard using a turbidimeter. Ten microliters of the donor strain suspension and 20 microliters of the recipient strain suspension were co-inoculated into 20 mL of Luria-Bertani broth and incubated overnight at 37°C with shaking. Following overnight incubation, 1 mL of the broth culture was diluted 10-fold with 100 μl solution of the diluted bacterial suspension were then plated onto a selection plate containing 700 μg/mL rifampicin and 1 μg/mL imipenem. The selection plate was incubated overnight at 37°C. Individual colonies that grew on the selection plate were isolated and identified using the EXS3600 automated microbial mass spectrometry system. Only colonies confirmed as *Escherichia coli* by mass spectrometry were further analyzed. These isolates underwent PCR, sequencing, and antimicrobial susceptibility testing to confirm successful plasmid conjugation.

#### 2.2.3 Preparation of bacterial DNA templates

Bacterial strains stored at ultra-low temperatures were revived by inoculation onto Columbia blood agar plates and incubated at 37°C for 24 hours. The following day, one to two well-isolated colonies were selected and transferred aseptically to sterile Eppendorf tubes containing 250 μL of nuclease-free water. After thorough vortexing, the suspensions were lysed by heat treatment at 100°C for 10 minutes in a heating block and immediately chilled on ice for 10 minutes. This cycle of heat treatment and chilling was repeated three times. Subsequently, cell debris was pelleted by centrifugation at 12,000 rpm for 15 minutes. The resulting supernatant, containing the bacterial DNA, was carefully transferred to a sterile 1.5 mL microcentrifuge tube and stored frozen for future use.

#### 2.2.4 Screening for virulence genes

A PCR-based method was employed to profile key virulence genes in hypervirulent *Klebsiella pneumoniae*. This included *rmpA*, a regulator of capsule production, and the *aerobactin* iron acquisition gene. Primer sequences were synthesized by Shanghai Shenggong Biological Engineering Co., LTD. Each PCR reaction contained 3 μL of DNA template, 1 μL each of upstream and downstream primers, 10 μL of Taq enzyme, and nuclease-free water added to a final volume of 25 μL. The thermal cycling conditions were: initial denaturation at 95°C for 5 minutes, followed by 35 cycles of denaturation at 95°C for 30 seconds, annealing at 55°C for 40 seconds, extension at 72°C for 30 seconds, and a final extension at 72°C for 5 minutes.

#### 2.2.5 Whole genome sequencing

CR-hvKP isolates identified as positive for *rmpA* and *aerobactin* genes underwent whole-genome sequencing (WGS) using the Illumina Novaseq 6000 platform. Bacterial genomic DNA was extracted beforehand with a Bacterial Genomic DNA Extraction Kit (Shanghai Shenggong). Following library construction, short reads were assembled with SPAdes software (v3.11.1). The assembled sequences were then analyzed on the Center for Genome Epidemiology (CGE) website for Multi-Locus Sequence Typing (MLST), plasmid incompatibility group typing (PlasmidFinder 2.1), and identification of antibiotic resistance genes (ResFinder 4.1). Kleborate v0.3.0 software was employed to search for virulence-related genes in *Klebsiella pneumoniae*. Additionally, the RAST server provided gene annotation. Plasmid mobility analysis was performed with oriTfinder software, and a synteny map was generated using Easyfig software. Notably, Shanghai OuYi Biomedical Technology Co., Ltd carried out the WGS procedure.

#### 2.2.6 Murine virulence experiment

Fifteen male BALB/c mice aged 6-8 weeks, weighing 22 ± 2 g, were randomly selected for this experiment. A hypervirulent *Klebsiella pneumoniae* strain, NTUH-K2044, was used to establish the positive control group, while *Klebsiella pneumoniae* ATCC700603 served as the negative control. The experimental procedure was as follows: single bacterial colonies were selected and cultured in 10 mL of Luria-Bertani broth. During the logarithmic growth phase, these colonies were collected, resuspended in solution, and adjusted to an OD_592_ of 1.0 (corresponding to approximately 1.0 × 10^8^ CFU/mL). The bacterial suspension was then diluted 1000-fold to a concentration of 1.0 × 10^5^ CFU/mL for intraperitoneal injection. Each mouse received an intraperitoneal injection of 200 μL of the diluted suspension. The mice were monitored continuously for 5 days. Following the observation period, the mice were euthanized, and liver tissues and peripheral blood were collected for further analysis. This study was approved by the Ethics Committee of Ningbo Medical Centre Lihuili Hospital (No. KY2022SL281-01).

#### 2.2.7 Mouse liver tissue hematoxylin and eosin staining

Liver tissues were collected and fixed in a 4% paraformaldehyde solution for 24 hours. Following fixation, tissues were dehydrated through a graded ethanol series (100%, 95%, 85%, 75%, 50%, and water). Subsequently, tissues were cleared in xylene for 20 minutes and embedded in paraffin at 58°C for 3 hours. Paraffin-embedded sections were then cut to a thickness of 5 μm. The sections were subsequently stained using hematoxylin for 1 minute, followed by rinsing in water and incubation in 0.1% sodium bicarbonate solution. Afterward, the sections were dehydrated through a graded ethanol series (85% and 95%) and stained with eosin for 10 seconds. Finally, the sections were dehydrated again (95% and 100% ethanol), cleared in xylene for 3 minutes, and mounted with neutral gum.

#### 2.2.8 Cytokine detection

ELISA assays were used to quantify the levels of three cytokines in mouse serum: tumor necrosis factor-alpha (TNF-α), interleukin-1β (IL-1β), and IL-6. Kits for these cytokines (Batch numbers: EK282HS for TNF-α, EK201B for IL-1β, and EK206 for IL-6; LinkedBio) were employed, and all procedures strictly adhered to the manufacturers’ instructions.

#### 2.2.9 Western blot for NF-kB signaling pathway detection

Liver tissues (100 mg) from each group were homogenized in 100 μL of RIPA lysis buffer (containing a 1% protease inhibitor cocktail, Batch number: AR0102, Boster, China) on ice for 30 minutes. The homogenate was then incubated at 100°C for 10 minutes to denature the proteins. Subsequently, 10 μg of denatured protein was loaded onto an SDS-PAGE gel and electrophoretically separated. The proteins were then transferred to a PVDF membrane (Millipore, USA). The membrane was blocked with 5% non-fat dry milk in tris-buffered saline (TBS) at room temperature for 1 hour. Subsequently, the membrane was incubated overnight at 4°C with primary antibodies specific for NF-κB p50 (Batch number: R1309-9, Hua’an Biology), NF-κB p65 (Batch number: ET1603-12, Hua’an Biology), phosphorylated NF-κB p65 (S529, Batch number: ET1604-27, Hua’an Biology), and GAPDH (Batch number: ET1601-4, Hua’an Biology). The membrane was then washed and incubated with horseradish peroxidase (HRP)-conjugated secondary antibody (Goat anti-Rabbit IgG-HRP, Batch number: HA1001, Hua’an Biology) at room temperature for 1 hour. Protein bands were visualized using a chemiluminescent detection system (Boster, China) and captured with a UVP protein imaging system (ChemiDoc-It Imaging System, USA). The intensity of each band was quantified using ImageJ software.

#### 2.2.10 Statistical analysis

Statistical analysis was carried out using SPSS (v22.0) and GraphPad Prism (v6.0). Continuous data were presented as mean ± standard deviation (x ± s). One-way analysis of variance (ANOVA) was employed for comparisons between multiple groups, with statistical significance defined as P < 0.05.

## 3 Results

### 3.1 Strain origin and patient demographics

Out of the 96 collected carbapenem-resistant *Klebsiella pneumoniae* strains, 70 were isolated from male and 26 from female patients. The mean age of the patients was 62 ± 15.5 years. The specimen types included sputum (62 strains, 64.58%), urine (14 strains, 14.58%), whole blood (8 strains, 8.33%), aspirate (2 strains, 2.08%), bile (2 strains, 2.08%), drain fluid (2 strains, 2.08%), feces (1 strain, 1.04%), and catheter tip (1 strain, 1.04%). Four strains originated from other specimen types.

Most CR-KP strains (n=43, representing 44.79%) were isolated from specimens obtained in the intensive care unit (ICU), followed by the neurosurgery department (n=31, 32.29%). Other departments contributing specimens included respiratory internal medicine (8 strains, 8.33%), nephrology (4 strains, 4.17%), hepatobiliary surgery (3 strains, 3.13%), infectious disease (3 strains, 3.13%), hematology (2 strains, 2.15%), and orthopedics (2 strains, 2.15%). More information on specimen types and the source of the 96 CR-KP strains collected is provided in **Supplementary Figures 1, 2**. In summary, our study identified sputum specimens as the most common source of CR-KP strains (64.58%), with the ICU department demonstrating the highest prevalence (44.79%). These findings align with previous research reporting a similar proportion of CR-KP strains isolated from sputum (59.2%) and a predominant distribution within the ICU setting (45.3%) (Shen et al., 2023). Therefore, strengthening CR-KP surveillance in clinical microbiology laboratories is crucial for preventing CR-KP infections and curbing the emergence of bacterial drug resistance.

### 3.2 Selection of hypervirulent carbapenem-resistant *Klebsiella pneumoniae*

**Figure 1:**
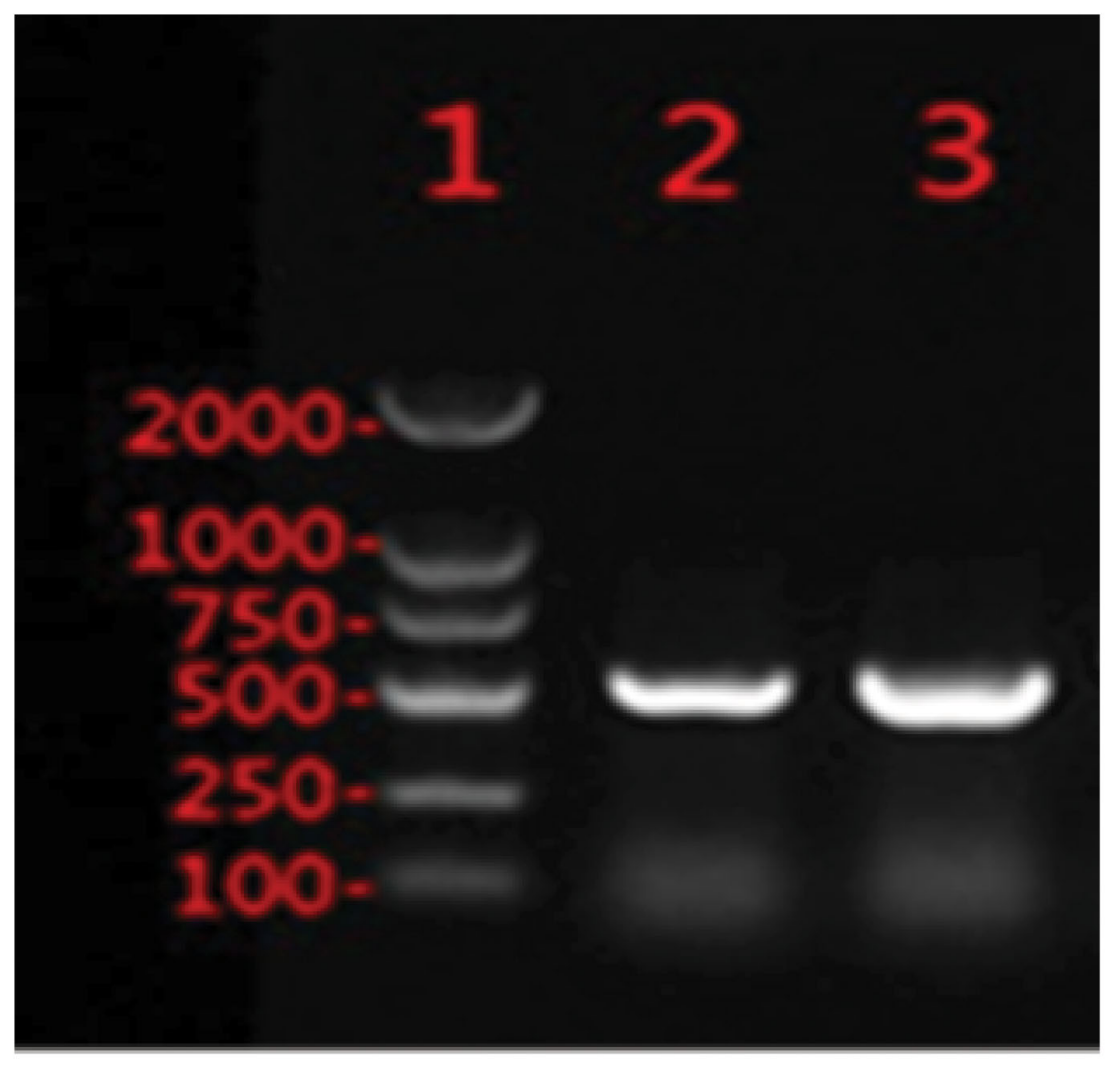
PCR Products of Virulence Genes. 1: Marker(100bp-2000bp); 2:*rmpA*; 3:*aerobactin*.

Applying the criteria for CR-hvKP outlined in reference (Bailey et al., 2016), PCR screening identified only a single strain, designated CR-hvKP57, positive for both the *rmpA* and *aerobactin* genes among the 96 collected carbapenem-resistant *Klebsiella pneumoniae* isolates. This strain, isolated from a sputum sample in the Department of Respiratory Medicine, yielded a detection rate of 1.04% (1/96). The primer sequences used are presented in **Table 1**, and the corresponding PCR products are shown in **Figure 1**.

**Table 1:** Primers for Virulence Genes.

Virulence genes	Primer sequence (5’-3’)	Fragment length (bp)
*rmpA*	F: ACTGGGCTACCTCTGCTTCA	536
	R: CTTGCATGAGCCATCTTTCA	
*aerobactin*	F: GCATAGGCGGATACGAACAT	556
	R: CACAGGGCAATTGCTTACCT	

### 3.3 Drug sensitivity results and plasmid conjugation experiment

The 96 CR-KP strains exhibited high resistance to most common antibiotics, except for polymyxin, tigecycline, and ceftazidime/avibactam. Notably, ceftriaxone, cefepime, and imipenem displayed a 100% resistance rate. Our findings align with previous research, where CR-KP strains demonstrated resistance exceeding 95% to cephalosporins and carbapenems (Zhang et al., 2021), and over 50% to fluoroquinolones and aminoglycosides (Cienfuegos-Gallet et al., 2022). The experimental strain, CR-hvKP57, remained susceptible to aztreonam, ciprofloxacin, levofloxacin, trimethoprim/sulfamethoxazole, amikacin, gentamicin, tobramycin, tigecycline, and ceftazidime/avibactam. However, it exhibited high-level resistance to all other tested antibiotics. Attempts to transfer the resistance plasmid were unsuccessful (**Table 2**).

**Table 2:** MICs of 96 isolates of carbapenem-resistant *Klebsiella pneumoniae* with CR-hvKP57 against antibiotics (μg/mL).

Antimicrobial Agent	MIC Breakpoints (μg/mL)			CR-KP		CR-hvKP57	
				(n=96)			
	S	I	R	resistant strains (n)	Percentage(%)	MIC	Drug-susceptibility results
ATM^a^	≤4	8	≥16	78	81.25	≤1	S
CRO^a^	≤1	2	≥4	96	100.00	>32	R
FEP^a^	≤2	–	≥16	96	100.00	>32	R
CIP^a^	≤0.25	0.5	≥1	70	72.92	0.25	S
LEV^a^	≤0.5	1	≥2	90	93.75	0.25	S
IMP^a^	≤1	2	≥4	96	100.00	>8	R
ETP^a^	≤0.5	1	≥2	73	76.04	>4	R
TZP^a^	≤8/4	–	≥32/4	81	84.38	>64	R
SXT^a^	≤2/38	–	≥4/76	79	82.29	≤1	S
NIT^a^	≤32	64	≥128	64	66.67	64	I
AMK^a^	≤4	8	≥16	54	56.25	≤2	S
GEN^a^	≤2	4	≥8	67	69.79	≤1	S
TOB^a^	≤2	4	≥8	66	68.75	≤1	S
POL^a^	–	≤2	≥4	7	7.29	4.00	R
TGC^b^	≤2	4	≥8	9	9.38	1.00	S
CZA^a^	≤8/4	–	≥16/4	8	8.33	3.00	S

Attempts to transfer the resistance plasmid via conjugation were unsuccessful. This is likely due to the absence of the origin of the transfer sequence (oriT) on the plasmid, which is essential for mobilization. Consequently, antibiotic susceptibility testing of the transconjugant could not be performed (**Table 2**). ATM, aztreonam; CRO, ceftriaxone; FEP, cefepime; CIP, ciprofloxacin; LEV, levoffoxacin; IMP, imipenem; ETP, ertapenem; TZP, piperacillin/tazobactam; SXT, trimethoprim/sulfamethoxazole; NIT, nitrofurantoin; AMK, amikacin; GEN, gentamicin; TOB, tobramycin; POL, polymyxin; TGC, tigecycline; CZA, ceftazidime-avibactam.

^a^CLSI refers to the Clinical and Laboratory Standards Institute, while ^b^FDA stands for the U.S. Food and Drug Administration. The “-” symbol indicates the absence of any relevant recommendations”.

### 3.4 Chromosomal genome features

**Figure 2:**
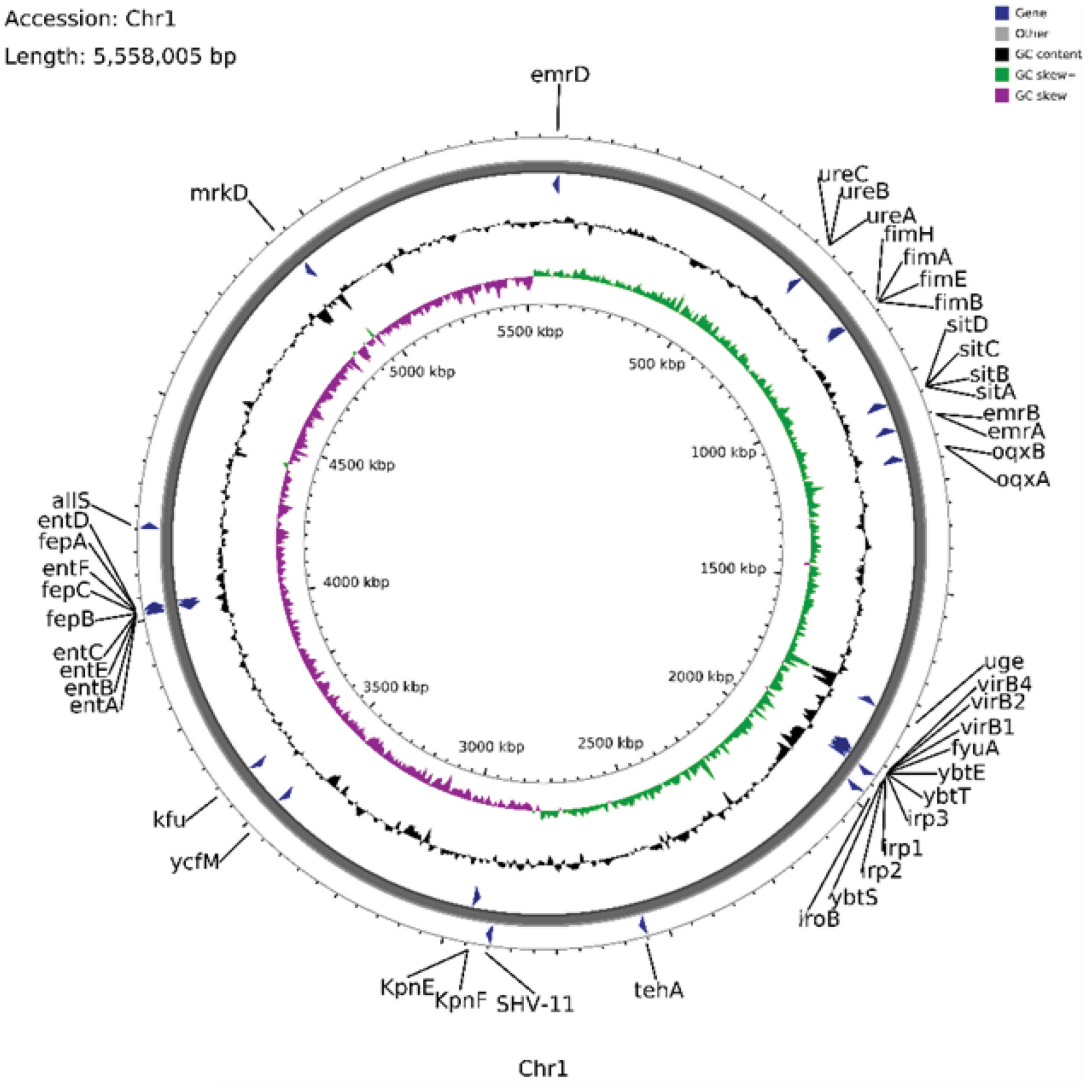
Annotation of the Chromosomal Genome Features, Some Virulence Genes, and Resistance Genes in CR-hvKP57 (Accession number: CP145493).

Following WGS of CR-hvKP57, analysis revealed a single chromosome of approximately 5,558,005 base pairs, designated CR-hvKP57-chromosome (Accession number: CP145493). MLST identified the strain as ST23 type, while *wzi* analysis confirmed it as the K1 capsular serotype. The chromosome harbored various resistance genes, including the broad-spectrum β-lactamase gene *bla*_SHV-11_, genes for MFS efflux pumps (*emrA*, *emrB*, *emrD*), and genes for RND efflux pumps (*oqxA*, *oqxB*). Additionally, the genome encoded multiple virulence factors categorized as: (1) adherence and pilus genes (Type I pili *fimABEH* and Type III pili *mrkD*), (2) iron acquisition genes (*Enterobactin* siderophore genes *entABCDE* and *entF*, iron transport proteins *fepABC*, *sitABC*), (3) siderophore genes (*Salmochelin iroB*), (4) *Yersiniabactin* genes (*ybtETS*, *irp1*, *irp2*), and (5) genes related to urease enzyme metabolism (urease accessory protein *ureABC*) (**Figure 2**).

### 3.5 Plasmid genome characteristics

Identified as a 230,808 bp fusion plasmid, Plasmid 1 belongs to the IncHI1B and IncFIBκ incompatibility groups and shares similarities with pLVPK-like virulence plasmids. Designated as pCR-hvKP57-plasmid 1 (accession number: CP145494), it harbors essential virulence genes associated with hypervirulent *Klebsiella pneumoniae*. These genes include: (1) *rmpA* and *rmpA2*, promoting capsule synthesis; (2) *iutA*, *iucABD*, involved in aerobactin virulence; (3) *iroBCN* and *irp5*, responsible for iron acquisition; (4) *virB* genes encoding the type IV secretion system; and (5) *pilW*, encoding a component for type IV pilus formation.

This study identified an 83,759-bp repB-type plasmid, designated pCR-hvKP57-plasmid 2 (accession number: CP145495). This plasmid harbors transposons IS*15* and IS*26*, along with conjugation-related proteins TraH and TraG. Notably, pCR-hvKP57-plasmid 2 lacks virulence and resistance genes.

**Figure 3:**
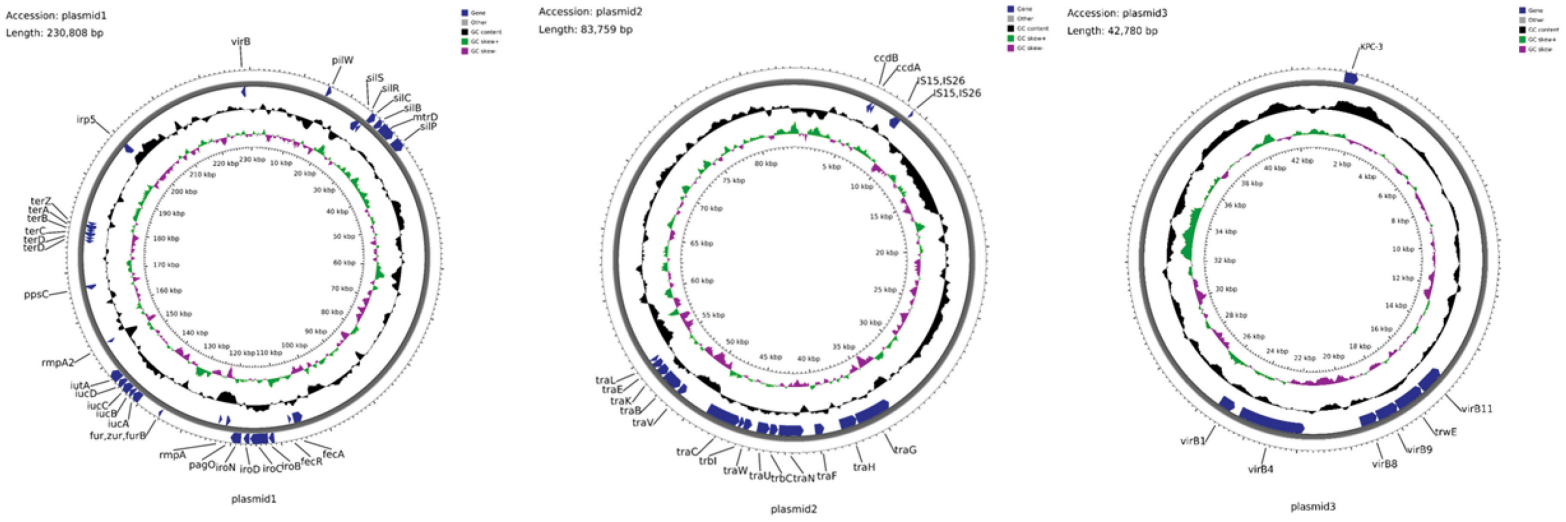
Annotation of the Plasmid Genome Features, Some Virulence Genes, and Resistance Genes in CR-hvKP57 (pCR-hvKP57-plasmid 1, accession number: CP145494; pCR-hvKP57-plasmid 2, accession number: CP145495 and pCR-hvKP57-plasmid 3, accession number: CP145496).

Designated as pCR-hvKP57-plasmid 3 (accession number: CP145496), plasmid 3 is a 42,780-base pair IncX5-type resistance plasmid that primarily harbors the carbapenem resistance gene *bla*_KPC-3_. Additionally, it encodes genes associated with the type IV secretion system (T4SS), including *virB1*, *virB4*, *virB8*, *virB9*, and *virB11* (**Figure 3**).

### 3.6 pLVPK-like virulence plasmid gene co-linearity analysis

**Figure 4:**
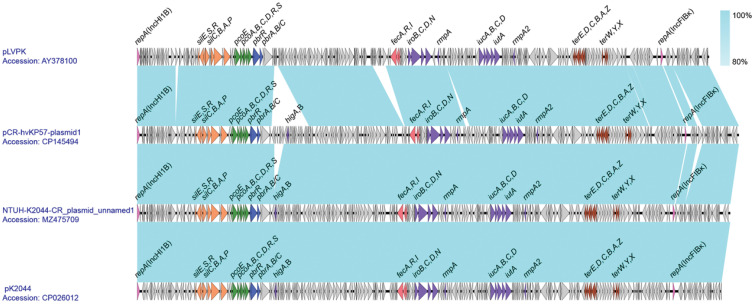
Comparative Genomic Analysis of Plasmid 1 genes (accession number: CP145494).

Analysis revealed that Plasmid 1 is a pLVPK-like virulence plasmid with a 50.17% GC content. It harbors two replicons, IncHI1B and IncFIBκ. Blastn comparison showed high similarity to *Klebsiella pneumoniae* plasmids NTUH-K2044-CR_unnamed1 (Accession number: MZ475709) and pK2044 (Accession number: CP026012). Plasmid 1 encodes regulatory genes (*rmpA* and *rmpA2*) for capsule synthesis and virulence. Additionally, it carries the aerobactin-encoding gene cluster (*iucABCD* and *iutA*) and the yersiniabactin-encoding gene cluster (*iroBCDN*), along with the *fecARI* gene cluster associated with iron uptake regulation. Furthermore, Plasmid 1 harbors various heavy metal resistance gene clusters, including those for potassium tellurite (terZABCDE and terXYW), lead (*pbrRABC* and *pbrAB*C), copper (*pcoEABCDRS*), and silver (*silCBAP* and *silRSE*), enhancing the strain’s environmental adaptability. Notably, compared to pLVPK, Plasmid 1 possesses an additional inserted segment downstream of the *pbr* gene cluster, containing the toxin-antitoxin system *higAB* (**Figure 4**).

### 3.7 KPC-3 resistance plasmid gene co-linearity analysis

**Figure 5:**
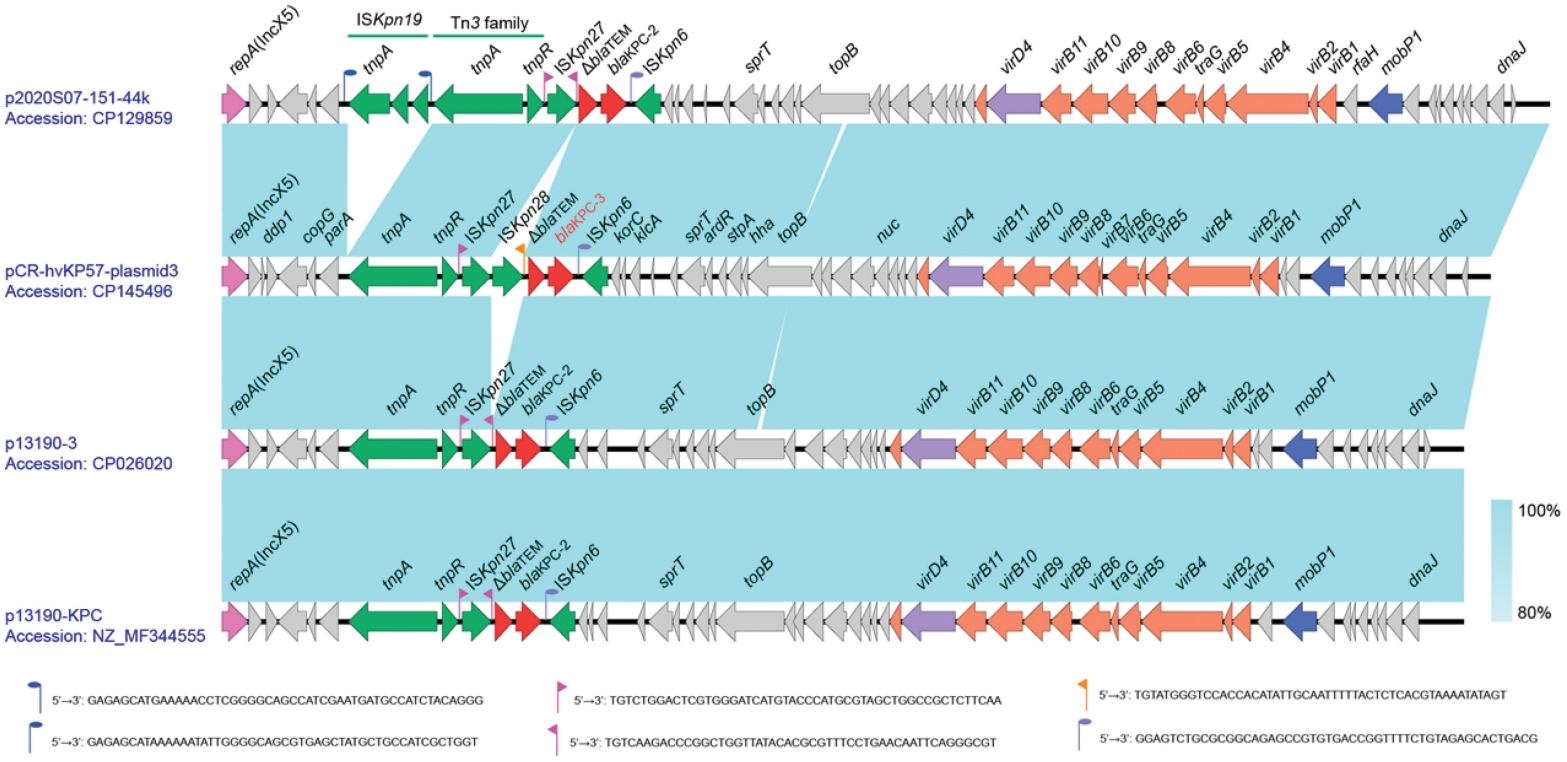
Comparative Genomic Analysis of Plasmid 3 Genes (accession number: CP145496).

Plasmid 3, with a GC content of 45.75%, is a resistance plasmid harboring the KPC-3 carbapenemase resistance gene. It carries only one carbapenemase resistance gene, *bla*_KPC-3_, alongside a truncated β-lactamase gene, Δ*bla*_TEM_. These genes reside within the IS element cluster/transposon region “*tnpA*-*tnpR*-*ISKpn27*-*ISKpn28*-Δ*bla*_TEM_-*bla*_KPC-3_-*ISKpn6*”. This arrangement resembles the typical NTEKPC-II structure, where KPC is located within a non-Tn4401 transposon. To further explore the genetic context of *bla*_KPC-3_, Plasmid 3 was compared to highly similar *Klebsiella pneumoniae* plasmids like p2020S07-151-44k (Accession number: CP129859), p13190-3 (Accession number: CP026020), and p13190-KPC (Accession number: NZ_MF344555). Notably, Plasmid 3 possesses an insertion of ISKpn28 upstream of the Δ*bla*_TEM_ gene, which is absent in the reference sequences. This insertion might lead to the loss of the downstream inverted repeat sequence (IRR) of ISKpn27. While Plasmid 3 encodes a relaxase (MobP1), an IV-type conjugative transfer protein (VirD4), and genes associated with the type IV secretion system (virB family), oriTfinder analysis suggests the absence of a critical transfer origin site (oriT) (**Figure 5**).

### 3.8 Mouse virulence experiment and HE staining of the liver

**Figure 6:**
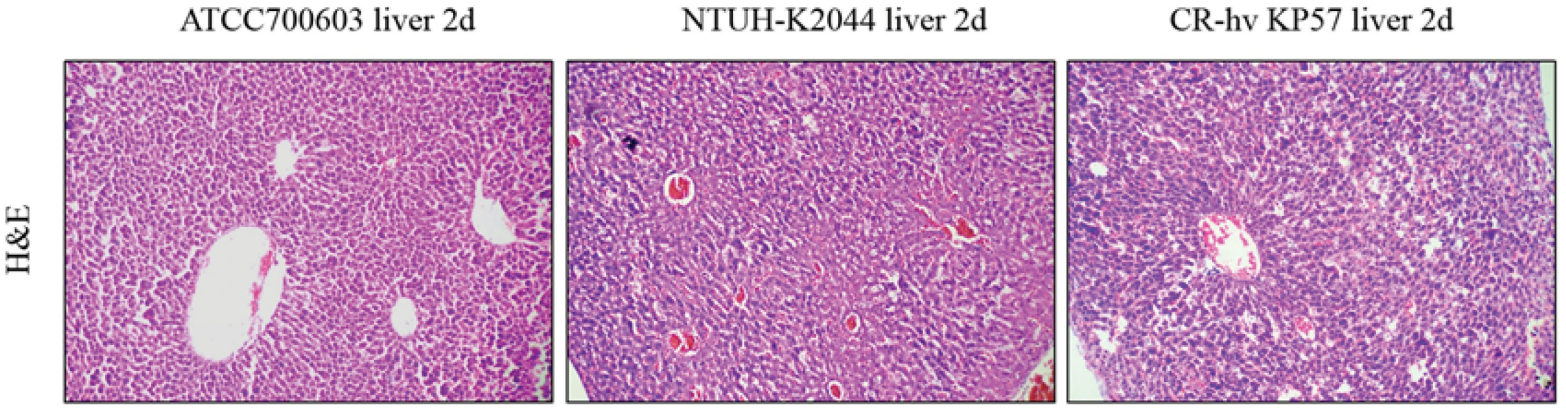
HE Staining of Liver Tissues from BalB/C Mice Infected with ATCC700603, NTUH-K2044, and CR-hvKP57 on Day 2.

On the first day post-injection, mice in the ATCC700603 group (negative control) displayed normal behavior. Conversely, mice in the NTUH-K2044 and CR-hvKP57 groups (positive controls) exhibited huddling behavior and lethargy following intraperitoneal administration of bacterial suspension (1.0 × 10^5^ CFU/mL). By the second day, mice in the ATCC700603 group maintained good health, while all mice in the NTUH-K2044 and CR-hvKP57 groups succumbed to infection. Histopathological analysis of liver tissue following HE staining revealed distinct findings across the three groups. The ATCC700603 group exhibited evidence of white blood cell infiltration and early liver abscess formation on day two. In contrast, the NTUH-K2044 group displayed liver hemorrhaging, hepatocyte nuclear loss, and widespread cell death. The CR-hvKP57 group showed liver hemorrhaging, partial hepatocyte nuclear loss, and vacuolation of liver cells on day two. These observations suggest that NTUH-K2044 and CR-hvKP57 infection resulted in high mortality rates, with acute liver injury identified as the likely cause of death (**Figure 6**). Notably, these findings differ from clinical observations, where hvKP strains are primarily associated with liver abscess formation in patients. Reported hvKP-induced liver abscesses typically involve community-acquired, antibiotic-sensitive *Klebsiella pneumoniae* (Celis et al., 2021). This contrasts with the current study’s experimental bacterium, CR-hvKP57, representing a hospital-acquired, carbapenem-resistant *Klebsiella pneumoniae* strain. Additionally, clinical data suggests a strong association between hvKP-mediated liver abscesses and underlying comorbidities in patients, such as diabetes and chronic diseases (Seetoh et al., 2013; Luo et al., 2014).

### 3.9 Detection of cytokines and NF-κB signaling pathway

**Figure 7:**
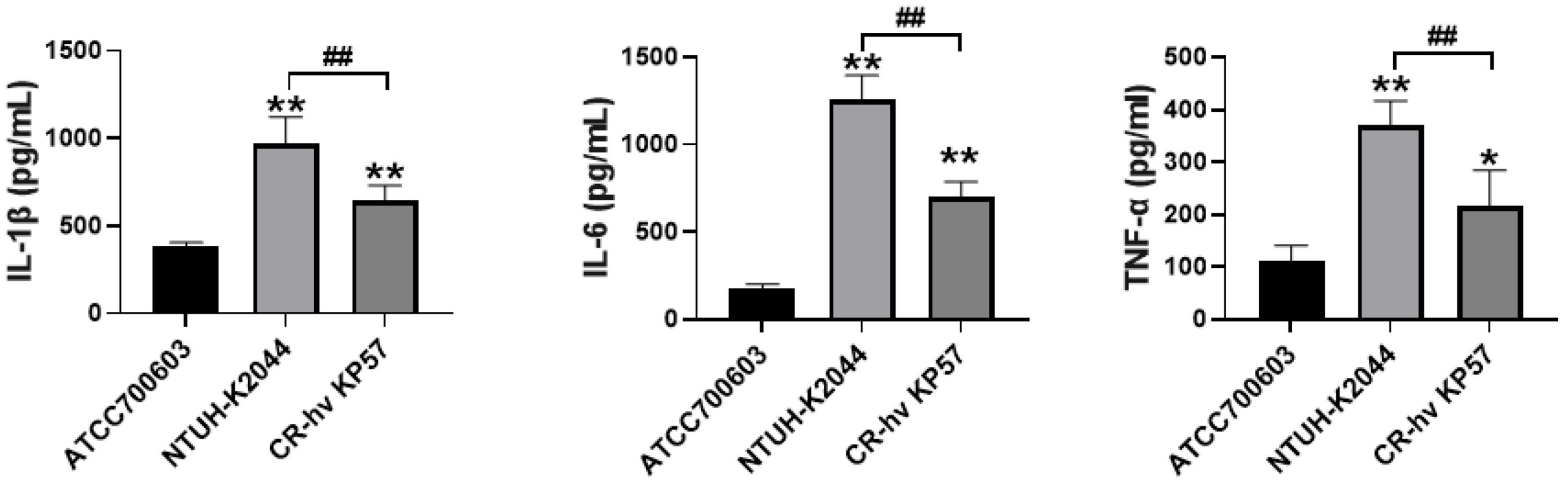
Mouse Serum Levels of IL-1β, IL-6, and TNF-α. Compared to the ATCC700603 group, ** *P*<0.01, * *P*<0.05; Compared to the NTUH-K2044 group, ## *P*<0.01.

Compared to the ATCC700603 group (negative control), the concentrations of IL-1β and IL-6 in the NTUH-K2044 and CR-hvKP57 groups (positive controls) were significantly elevated (*P* < 0.01). However, the level of TNF-α displayed the most significant increase in the NTUH-K2044 group (*P* < 0.01). While the CR-hvKP57 group also showed a significant increase in TNF-α compared to the ATCC700603 group (*P* < 0.05), the concentrations of IL-1β, IL-6, and TNF-α in the CR-hvKP57 samples were significantly lower than those observed in the NTUH-K2044 group (*P* < 0.01) (**Figure 7**).

**Figure 8:**
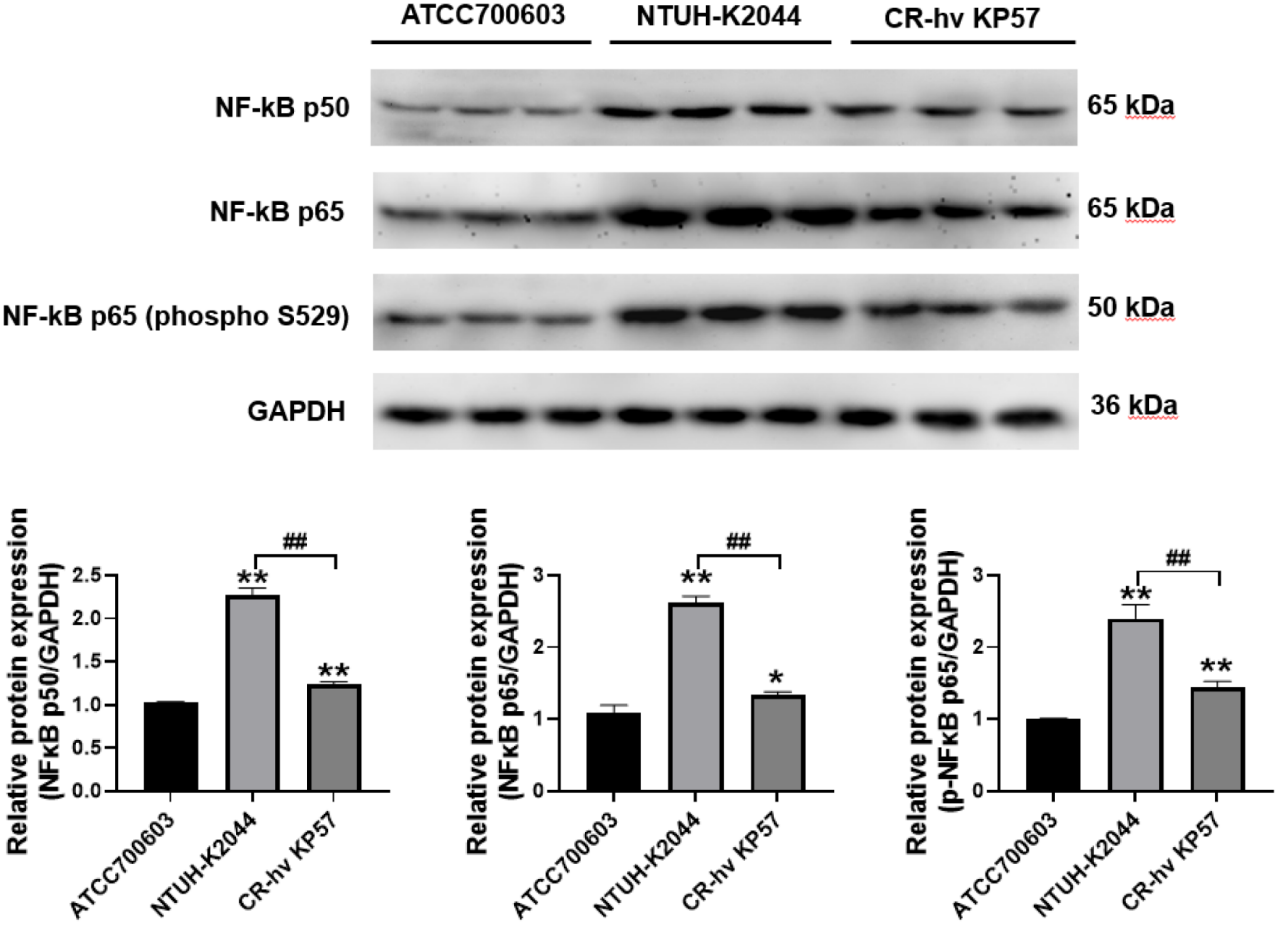
Expression Levels of NF-κB p50, NF-κB p65, and Phosphorylated NF-κB p65 (phospho S529) in Mouse Liver Tissues. Compared to the ATCC700603 group, ** P<0.01, * P<0.05; Compared to the NTUH-K2044 group, ## P<0.01.

Western blot analysis revealed upregulated expression of NF-κB p50 and phosphorylated NF-κB p65 (phospho S529) in both NTUH-K2044 and CR-hvKP57 samples compared to ATCC700603 samples (*P* < 0.01). Notably, NF-κB p65 expression was significantly higher in NTUH-K2044 samples than in ATCC700603 samples (*P* < 0.01). However, the increase in these proteins was less pronounced in the CR-hvKP57 group, though a significant difference remained (*P* < 0.05). Furthermore, CR-hvKP57 exhibited a significant decrease in the expression of NF-κB p50, NF-κB p65, and phosphorylated NF-κB p65 (phospho S529) compared to NTUH-K2044 (*P* < 0.01). These observed changes in expression align with the trends noted in cytokine expression (**Figure 8**).

## 4 Discussion

Several studies have identified virulence factors associated with CR-KP, including *magA*, *rmpA*, *wabG*, *uge*, *entB*, *ycfM*, *kpn*, *wcaG*, *fimH*, *mrkD*, and *iutA* (Xie et al., 2021; Jiang et al., 2024). Notably, *wabG*, *uge*, *ycfM*, and *entB* exhibit high carriage rates of 88%, 86%, 80%, and 72%, respectively (Candan and Aksöz, 2015). The virulence plasmid of hvKP can be transferred horizontally to CR-KP strains independently or through conjugation with the IncF plasmid, potentially leading to the formation of CR-hvKP strains (Xu et al., 2021). Acquisition of the virulence plasmid equips these CR-hvKP strains with enhanced virulence and a heightened capacity for transmission, increasing the risk of hospital and community outbreaks (Tian et al., 2021; Xie et al., 2021). The widespread dissemination of carbapenemase-encoding plasmids has further contributed to the recent rise in CR-hvKP reports (Wang et al., 2022). The first documented cases of CR-hvKP were reported by Yao et al. in the Beijing region, involving four ST11-type strains responsible for hospital outbreaks with a concerningly high mortality rate (Yao et al., 2015). Subsequently, in 2021, Zhou et al. isolated 16 CR-hvKP strains from a total of 1081 *Klebsiella pneumoniae* isolates in Shanghai, representing a prevalence of 1.48% (Zhou et al., 2021b). A multicenter epidemiological analysis by Zhang et al. identified the ST11-K64 strain harboring the KPC-2 carbapenemase gene as the most prevalent CR-hvKP type (Zhang et al., 2020). This strain’s prevalence has risen from 2.1% to 7.0%, with the highest concentrations observed in the Henan and Shandong regions. This study, conducted in Ningbo, China, identified a single CR-hvKP strain among 96 carbapenem-resistant *Klebsiella pneumoniae* isolates, yielding an isolation rate of 1.04%. This rate aligns with the findings reported in the literature (Zhou et al., 2021a). However, whole-genome sequencing revealed that this strain belonged to the ST23-K1 type and harbored the KPC-3 carbapenemase gene, differentiating it from the ST11-K64 type commonly described in other studies (Liu et al., 2022b). The ST11-K64 lineage, carrying the *blaKPC-2* gene, represents a dominant and widely distributed strain, particularly prevalent in China (Yang et al., 2020; Wang et al., 2023). While a single prior report documented an NDM-1-producing, serotype K1 ST23 hypervirulent *Klebsiella pneumoniae* strain in China (Liu and Su, 2019), no previous reports have identified KPC-3-producing ST23-K1 CR-hvKP strains. Subsequent whole-genome assembly analysis revealed the presence of three plasmids within this CR-hvKP strain. Notably, plasmid 1 was identified as a pLVPK-like virulence plasmid, harboring the two replication genes *IncHI1B* and *IncFIBκ*, characteristic of hypervirulent *Klebsiella pneumoniae* of the KPVP-1 type. Plasmid pLVPK (Accession number: AY378100) serves as an example of this plasmid class (Harada et al., 2019). Plasmid 1 additionally harbors the *rmpA* and *rmpA2* genes, critical virulence factors responsible for the hypermucoviscous phenotype in bacteria (Hsu et al., 2011; Shoja et al., 2022). Importantly, these genes contribute to the bacteria’s resistance to phagocytosis by host neutrophils (Lin et al., 2019). Furthermore, plasmid 1 harbors the *iucABCD* and *iutA* gene clusters encoding *aerobactin* alongside the *iroBCDN* gene cluster associated with *salmochelin* production. Collectively, these genes enhance the bacteria’s ability to acquire essential iron from the host environment, promoting its survival (Bailey et al., 2016). The presence of all these genetic elements aligns with the observed high virulence of the bacteria (Kocsis, 2023; Chen et al., 2023b). Plasmid 3 of this strain harbors only one carbapenem-resistance gene, *bla*_KPC-3_, and a truncated β-lactamase gene, Δ*bla*_TEM_. These genes reside within an IS element cluster/transposon structure designated “*tnpA-tnpR-ISKpn27-ISKpn28-ΔblaTEM-blaKPC-3-ISKpn6*”. This arrangement exhibits some resemblance to the typical NTEKPC-II structure, where the KPC gene is located on a non-Tn4401 transposon, contrasting with the more widely distributed *bla*_KPC_ often found on the Tn4401 transposon, as demonstrated in the literature (Cuzon et al., 2011). Notably, oriTfinder analysis suggests this plasmid lacks the critical transfer origin site (oriT). This absence theoretically renders it incapable of autonomous plasmid transfer between bacterial cells (Carballeira et al., 2014; Traore et al., 2018), which aligns with the multiple failed attempts at plasmid conjugation transfer observed in our study.

Tang et al. demonstrated the crucial role of pLVPK in determining *Klebsiella pneumoniae* virulence. Strains harboring the “*terW-rmpA-iutA-silS*” locus derived from pLVPK have been associated with increased susceptibility to liver abscess formation in patients (Tang et al., 2010). However, in our *in vivo* mouse virulence experiments, where a bacterial suspension of 1.0 × 10^5^ CFU/mL was administered intraperitoneally, both the NTUH-K2044 and CR-hvKP57 groups exhibited rapid mortality within 2-3 days across repeated trials in contrast to the ATCC700603 group, which developed liver abscesses by day 5. Pathological examination revealed acute liver damage as the primary cause of death in these mice. Our findings indicated significantly elevated levels of IL-1β, IL-6, and TNF-α in the NTUH-K2044 and CR-hvKP57 samples compared to the ATCC700603 group. IL-1β, the predominant proinflammatory cytokine within the IL family, exists in two isoforms: IL-1α and IL-1β. Notably, IL-1β plays a central role in acute inflammation (Kwak et al., 2016). IL-1α and IL-1β, with IL-1β playing a central role in acute inflammation (Weber et al., 2010a). IL-6, a pleiotropic cytokine secreted by macrophages, T cells, and B cells, is known to be involved in immune response regulation and inflammatory processes (Baran et al., 2018). The observed increase in IL-1β, IL-6, and TNF-α levels suggests activation of the NF-κB signaling pathway (Hu et al., 2014). NF-κB, named for its specific binding to the κ light chain immunoglobulin enhancer in B lymphocytes (Hu et al., 2014), is activated through post-translational modifications like phosphorylation (such as phosphorylation) (Pan et al., 2017). Activated NF-κB dimers, p50, and p65 translocate to the nucleus, triggering the reactivation of downstream proinflammatory cytokines such as IL-1, IL-6, and IL-8 (Perkins, 2015; Giridharan and Srinivasan, 2018). These cytokines, in turn, can activate the NF-κB signaling pathway (Weber et al., 2010b), forming a positive feedback loop that leads to a cytokine cascade and inflammatory response (Mudd et al., 2021; Liang et al., 2021). Western blot experiments in our study corroborated these observations in the NTUH-K2044 and CR-hvKP57 groups, demonstrating a significant increase in the expression of NF-κB p50, NF-κB p65, and phosphorylated NF-κB p65 (phospho S529). Based on these findings, it is highly conceivable that the upregulation of these cytokines and the subsequent inflammatory storm are the primary causes of acute liver necrosis in the infected mice. Therefore, anti-cytokine therapy targeting IL-1, IL-6, and TNF-α antagonists may be a promising strategy to mitigate the hyperinflammatory state associated with CR-hvKP-induced inflammatory storm syndrome. The use of TNF-α inhibitors, such as elanercept and infliximab, could be potential candidates for further investigation (Yao et al., 2021; Zhou et al., 2021b).

## 5 Conclusions

In summary, this study identified a strain of ST23-K1 type CR-hvKP carrying the KPC-3 carbapenemase gene in China’s Ningbo region. This strain exhibits significant differences from previously reported ST23-K1 *Klebsiella pneumoniae* strains carrying other resistance genes, such as extended-spectrum β-lactamases, NDM-1, and *OXA-48* (Cheong et al., 2016; Wei et al., 2016; Cheong et al., 2017; Liu and Su, 2019; Li et al., 2020; Blanc et al., 2021). Furthermore, mouse virulence experiments demonstrated a notable increase in mortality rates linked to activation of the NF-κB signaling pathway, leading to a cytokine storm. A retrospective investigation of the patient’s case revealed the detection of the pathogen in a sputum specimen 3 days after admission, leading to a diagnosis of hospital-acquired pneumonia. *In vitro* susceptibility testing demonstrated sensitivity to ceftazidime/avibactam. This led to treatment with intravenous ceftazidime/avibactam (2.5g every 8 hours) for 6 days, resulting in successful resolution. Subsequent testing of the sputum specimen did not detect the presence of the pathogen again. The consistent *in vitro* susceptibility profile can be attributed to avibactam’s effective inhibition against Ambler class A enzymes and its correlation with the production of the KPC-3 carbapenem-resistant gene. The emergence of CR-hvKP is currently attributed to two primary mechanisms: first, the acquisition of carbapenem resistance genes by hvKP through plasmids, and second, CR-hvKP obtaining virulence plasmids that contain genes responsible for increased virulence factors (Tang et al., 2020; Xie et al., 2021; Han et al., 2022; Li et al., 2023). Accordingly, the CR-hvKP57 strain identified in this study has evolved from the simultaneous acquisition of plasmids carrying carbapenem resistance genes and super-virulence genes. These findings underscore the necessity of judicious antibiotic utilization to prevent the rise of bacterial resistance in the Ningbo area. Moreover, it is imperative to enhance infection control strategies to mitigate the concurrent dissemination of virulence and resistance plasmids within the region.

## Data Availability

The datasets presented in this study can be found in online repositories. The names of the repository/repositories and accession number(s) can be found in the article/**Supplementary Material**.
