# Supplementary material for: Molecular characteristics and pathogenic mechanisms of KPC-3 producing hypervirulent carbapenem-resistant Klebsiella pneumoniae (ST23-K1)
Source: Front Cell Infect Microbiol. 2024 Aug 15;14:1407219. doi: 10.3389/fcimb.2024.1407219 (PMC11358127; doi:10.3389/fcimb.2024.1407219)

**Supplemental material**

**Supplementary Figures 1**

Sample Types of 96 carbapenem-resistant Klebsiella pneumoniae Strains

**
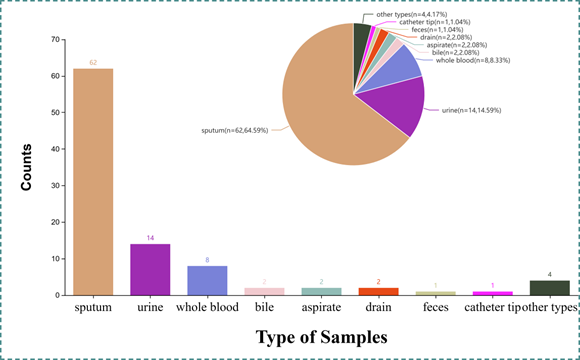
**

**Supplementary Figures 2**

Source of 96 carbapenem-resistant Klebsiella pneumoniae Samples.


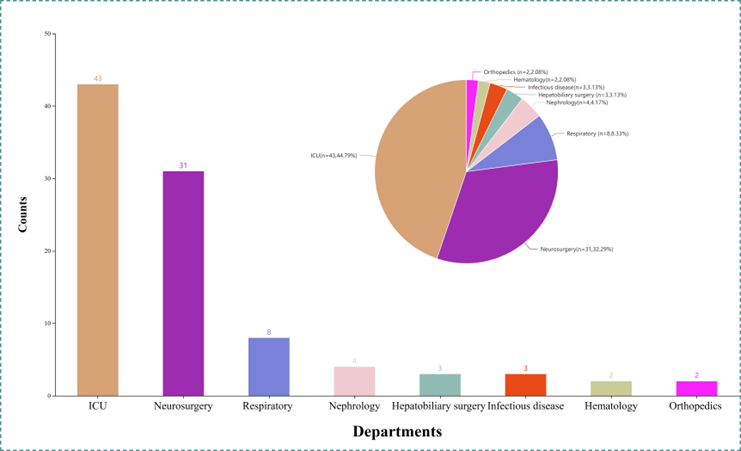

Supplement: Supplementary file 1 [file Table1.docx]
